# Co-delivery of 5-fluorodeoxyuridine and doxorubicin via gold nanoparticle equipped with affibody-DNA hybrid strands for targeted synergistic chemotherapy of HER2 overexpressing breast cancer

**DOI:** 10.1038/s41598-020-79125-0

**Published:** 2020-12-16

**Authors:** Chao Zhang, Fanghua Zhang, Mengnan Han, Xuming Wang, Jie Du, Honglei Zhang, Wei Li

**Affiliations:** College of Chemistry and Environmental Science, Key Laboratory of Chemical Biology of Hebei Province, Laboratory of Medicinal Chemistry and Molecular Diagnosis of the Ministry of Education, Hebei University, Baoding, 071002 China

**Keywords:** Biological techniques, Cancer, Nanoscience and technology

## Abstract

Combination chemotherapy is still of great importance as part of the standard clinical care for patients with HER2 positive breast cancer. As an attractive component, gold nanoparticles (AuNPs) have been extensively studied as biosafety nanomaterials, but they are rarely explored as drug nanocarriers for targeted co-delivery of multiple chemotherapeutics. Herein, a novel affibody-DNA hybrid strands modified AuNPs were fabricated for co-loading nucleoside analogue (5-fluorodeoxyuridine, FUdR) and anthracycline (doxorubicin, Dox). FUdRs were integrated into DNA hybrid strands decorated on AuNPs by DNA solid phase synthesis, and Dox molecules were intercalated into their duplex regions. Affibody molecules coupled to the DNA hybrid strands were distributed the surface of AuNPs, giving them targeting for HER2. The new dual-drug-containing affibody-DNA-AuNPs (Dox@affi-F/AuNPs) owned compact and stable spherical nanostructures, and precise drug loading. Cytotoxicity tests demonstrated that these nanoparticles caused a higher inhibition in HER2 overexpressing breast cancer cells, and showed better synergistic antitumor activity than simple mixture of the two drugs. The related mechanistic studies proved that Dox@affi-F/AuNPs achieved a remarkable combined antitumor activity of Dox and FUdR by promoting more cells to enter apoptosis pathway. Our work provided a nanomedicine platform for targeted co-delivery of nucleoside analog therapeutics and anthracycline anticancer drugs to achieve synergistic treatment of HER2^+^ cancer.

## Introduction

Breast cancer is a common malignant tumor with the highest incidence among women in developing countries, and also the leading cause of cancer-related deaths for women around the world^1,2^. Approximately, 15% to 20% of breast cancer patients are identified as HER2-positive (HER2^+^) cases^3^. According to the clinical guideline, antibody therapies against HER2 (Trastuzumab, Pertuzumab and Ertumaxomab) become a standard treatment strategy for HER2^+^ breast cancer. Although monoclonal antibodies significantly prolonged the overall survival of patients in clinics, they also have some limitations, including the limited ability to penetrate cells and tissues, the possibility of causing an immune response, and the high cost^4^. Moreover, 40–60% of HER2^+^ breast cancer patients do not respond to the treatment or develop primary and secondary drug resistance to antibody therapy^5,6^. To address these issues, many researchers have developed small peptide mimics of antibodies against HER2, such as affibody molecules, and exploited them for preparing various drug delivery systems to achieve targeted therapy for HER2^+^ breast cancer^7–9^. In comparison with full antibodies (~ 150 kDa), HER2-binding affibody molecules have smaller size (~ 7.0 kDa), more well-defined structure, easier site-specific modification, and nanomolar to picomolar antigen-binding affinity. More importantly, they can be produced efficiently by engineering bacteria, which greatly reduces production costs.

In addition to antibody therapy, chemotherapy is still the most widely received anti-cancer treatment strategy for HER2^+^ breast cancer^10–12^. In particular, combination chemotherapy is considered as an effective approach, because it can improve response rate, overcome drug-resistance and reduce side effects^13,14^. For example, anthracycline chemotherapeutics (doxorubicin, daunorubicin and aclarubicin) are often used in combination with nucleoside analogue (5-fluorouracil, 5-FU) for postoperative adjuvant chemotherapy and preoperative neoadjuvant chemotherapy of breast cancer, and show good efficacy in preliminary clinical application^15^. However, the conventional “cocktail”-based drug mixtures lead to different pharmacokinetic characteristics of the co-applied drugs, resulting in inconsistent drug absorption at the tumor site and insufficient treatment effect^16^. Thus, many drug delivery systems, such as liposomes^17^, nanomicelles^18^ and nanoparticles^19^, have been designed and prepared for drug co-loading in cancer combination therapy. Although these nanoscale carriers are often effective at noncovalent encapsulation of hydrophobic drugs, the encapsulation of hydrophilic drugs may result in poor loading and low drug encapsulation efficiency. Therefore, there is an urgent need to develop an improved drug loading strategy for the delivery of hydrophilic chemotherapeutic agents.

Nowadays, DNA has attracted more and more attention as a promising building block for nano-drug loading devices because of its programmability, predictability and biocompatibility^20^. Moreover, various chemotherapeutic drugs can be easily covalently integrated or non-covalently incorporated into DNA strands, such as 5-fluorodeoxyuridine (FUdR, metabolite of 5-FU)^21–25^ and doxorubicin (Dox)^26–29^. However, DNA strands alone are insufficient to be used as a drug delivery vehicle due to its lack of functionality. Recently, combining DNA strands with other nanomaterials and giving them functionality are an attractive research direction. Among them, as excellent functional materials, gold nanoparticles (AuNPs) with their functional versatility, biocompatibility, low/non-toxicity are widely applied to construct hybrid system with DNA for biomedical application, in which the Au-thiol interaction facilitates simple DNA functionalization on the AuNPs surface^30,31^. Many studies^32,33^ have demonstrated that DNA-functionalized AuNPs can be readily taken up by cells and are of high stability in buffer or serum-containing solution, which supports the DNA-AuNPs as a potential building material for the establishment of nanocarriers. However, there are few reports of using DNA-AuNPs for co-loading of multiple chemotherapeutics.

**Scheme 1:**
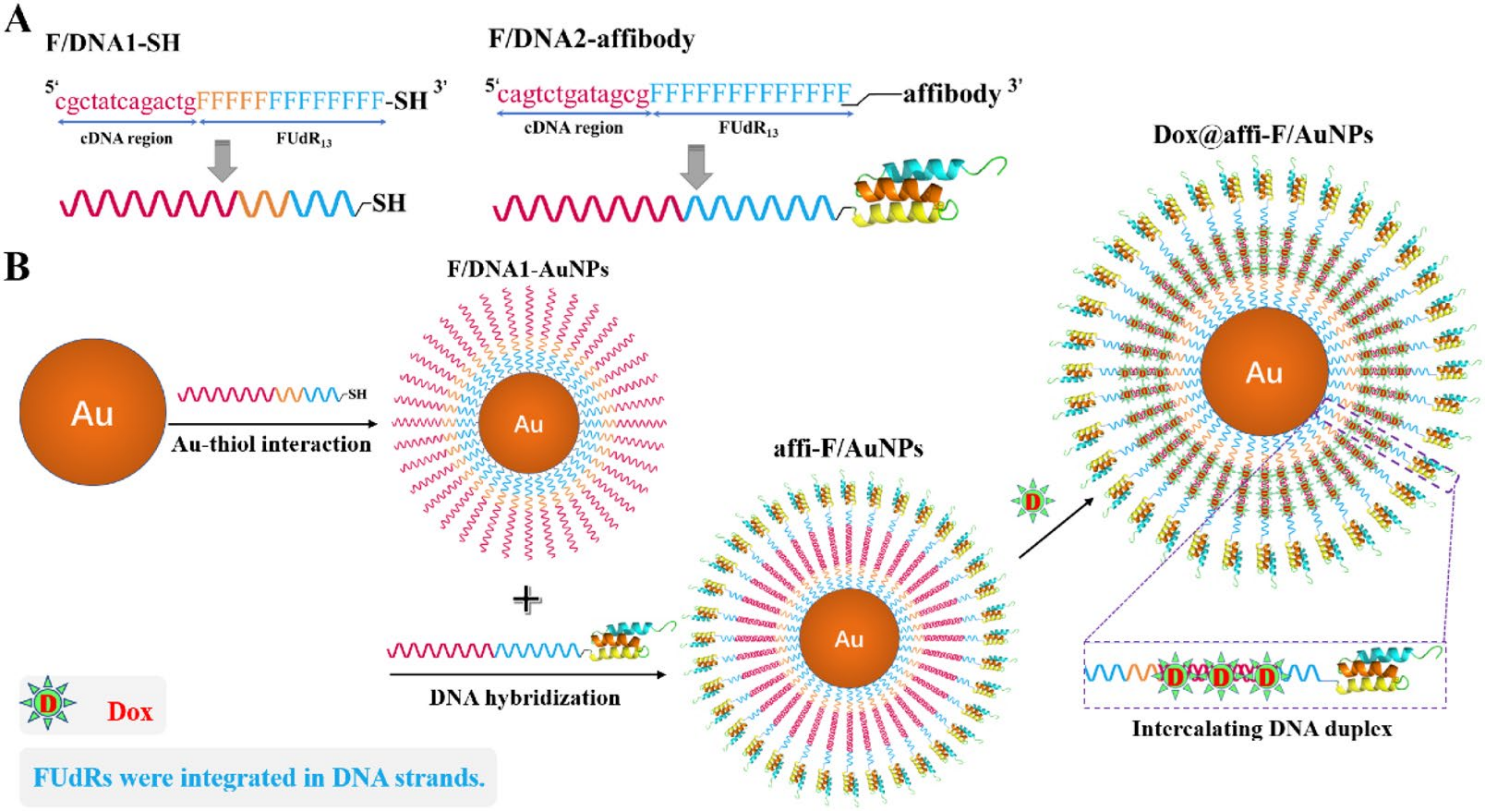
**(A**) Design of FUdR-containing DNA strands for construction of affi-F/AuNPs. (**B**) Schematic illustration of preparation process of Dox@affi-F/AuNPs.

In the present study, the DNA-AuNPs were employed as nanocarriers for co-loading FUdR and Dox, and conjugated them with affibody molecule to achieve HER2-targeted treatment in breast cancer. As shown in Scheme 1A, 13 FUdR molecules were respectively connected to the 3′-ends of the two DNA strands one by one through the DNA solid-phase synthesis method, forming FUdR-DNA strands. The 3′-end of one of the two FUdR-DNA strands was modified with a thiol group (named F/DNA1-SH) for surface functionalization of AuNPs. An affibody molecule was attached to the 3′-end of the other FUdR-DNA strand (named F/DNA2-affibody), which specifically bound HER2 on the surface of cancer cells. Subsequently, AuNPs were modified with F/DNA1-SH through Au-thiol bond formation, followed by hybridization with F/DNA2-affibody to form the affibody-FUdR-DNA hybrid strands decorated AuNPs (affi-F/AuNPs). Dox can be effectively loaded into DNA duplex of affi-F/AuNPs by intercalation (Scheme 1B). As a result, affi-F/AuNPs co-loaded with FUdR and Dox were obtained and designated as Dox@affi-F/AuNPs. To evaluate the synergistic therapeutic effect of FUdR and Dox, the targeted uptake of Dox@affi-F/AuNPs and their selective inhibitory activity on HER2^+^ breast cancer cells were investigated. Furthermore, the cell apoptosis was determined to explore the anticancer mechanism of Dox@affi-F/AuNPs. The affibody modified DNA-AuNPs may serve as a prospective targeted drug co-delivery system for the combination therapy of nucleoside analogues and the DNA-interacted anthracycline anticancer drugs.

## Materials and methods

### Materials and chemicals

5-Fluorodeoxyuridine, doxorubicin hydrochloride (Dox·HCl), Gold chloride hydrate (HAuCl_4_), sodium citrate tribasic dihydrate were obtained from Macklin Inc. (Shanghai, China). *N*-(ε-maleimidocaproyloxy)sulfosuccinimide ester (Sulfo**-**EMCS) were purchased from Aladdin Reagent Database Inc. (Shanghai, China). 4,6-Diamidino-2-phenylindole (DAPI) were obtained from Sangon Biotech (Shanghai) Co., Ltd. Trypsin, 3-(4,5-dimethylthiazol-2-yl)-2,5-diphenyl-2H-tetrazolium bromide (MTT), antibiotic–antimycotic (100 ×) and fetal bovine serum (FBS) were purchased from Wisent Biotechnology (Nanjing) Co. Ltd (Nanjing, China). All chemicals were used without further purification.

### Solid-phase synthesis of F/DNA1-SH and F/DNA2-NH_2_

The FUdR-containing oligonucleotides were synthesized on a Dr. Oligo 96 Oligo Synthesizer (Biolytic Lab Performance, Inc. Fremont, USA) using the standard solid-phase phosphoramidite methodology. Bases and reagents were purchased from Glen Research (Sterling, VA, USA). FUdR phosphoramidite was obtained from our lab^28^. To incorporate the drug into DNA strands, FUdR and commercial A, T, C, G phosphoramidite monomers were used to synthesize the FUdR-containing strands. The oligonucleotide used to functionalize the AuNPs was 3′thiol-modified FUdR-containing strand (F/DNA1-SH, sequence information was listed in Table S1). The oligonucleotide used to link targeted ligand was 3′ amine-functionalized FUdR-containing strand (F/DNA2-NH_2_, sequence information was listed in Table S1). The obtained F/DNA1-SH and F/DNA2-NH_2_ were purified by reverse-phase high-performance liquid chromatography (RP-HPLC) and characterized by LCMS-2020 (Shimadzu, Japan). The DNA concentration was determined by monitoring the absorbance at 260 nm with Nanodrop 2000c (Thermo Fisher Scientific, USA). For quantification of FUdR-DNA strands loaded on the gold nanoparticle and cellular imaging, an additional 5-carboxyfluorescein (FAM)-modified strand (FAM-F/DNA1-SH, listed in Table S1) was synthesized and purified according to conventional approach.

### Synthesis and purification of F/DNA2-affibody

The sequence of affibody molecule Z_hcHER2:342_ used in this study was MIHHHHHHLQVDNKFNKEMRNAYWEIALLPNLNNQQKRAFIRSLYDDPSQSANLLAEAKKLNDAQAPKVDC (71aa, Molecular weight: 8331.42 Da).

The synthesis process of F/DNA2-affibody was shown in Scheme S1. In a typical reaction, F/DNA2-NH_2_ (81.8 µg, 10 nmol) in 200 µL of phosphate-buffered saline (PBS) was added to 500 µL of 10 times molar excess of Sulfo-EMCS in HEPES buffer (0.1 M, pH 7) and incubated at 37 °C for 3 h. Then, F/DNA2-Sulfo-EMCS was obtained by ethanol precipitation, and dissolved in 100 μL of PBS. The F/DNA2-Sulfo-EMCS solution was treated with 108 μg (13 nmol) affibody dissolved in 500 μL of PBS buffer for overnight at room temperature. Then, the reaction mixture was purified on a Capto DEAE column (1 mL, GE Healthcare) and HisTrap HP column (1 mL, GE Healthcare)^34^. Finally, the product was analyzed and characterization by 2% agarose gel electrophoresis and 10% SDS–polyacrylamide gel electrophoresis (SDS-PAGE). The pure F/DNA2-affibody was concentrated using Amicon ultracentrifugal filters (MWCO 10 kDa) and stored at 4 °C.

### Synthesis of AuNPs

Citrate-capped AuNPs were synthesized by the reduction of Au ion with citric acid at high temperature^35^. 50 mL of HAuCl_4_ aqueous solution (0.01% by weight) was heated to 110–120 °C. Then, 500 μL of sodium citrate solution (1% by weight) was quickly added with stirring. When the color of the solution changed from light yellow to dark red (about 2 min), the reaction stopped. The solution was cooled down at room temperature and stored at 4 °C.

### Preparation of affi-F/AuNPs and Dox@affi-F/AuNPs

The disulfide bond in F/DNA1-SH (50 μL, 100 μM) was reduced to monothiol by dithiothreitol (DTT, 100 folded excess) in ddH_2_O at 37 °C for 2 h. Then, the freshly reduced F/DNA1-SH strands were added dropwise to 3 nM AuNPs (5 mL). After further shaking for 16 h, a salt aging process was used for 8 h to gradually reach a final 0.1 M NaCl solution by adding 2 M NaCl. The solutions were incubated for a further 16 h with shaking, and purified through multiple centrifugation-washing methods. Then, the obtained F/DNA1-modified AuNPs (F/DNA1-AuNPs) were mixed with F/DNA2-affibody, and the mixture was annealed in phosphate buffer to construct affi-F/AuNPs. The obtained affi-F/AuNPs were analyzed by 2% agarose gel electrophoresis. The gel was run at 100 V for 1 h and stained with GelStain. Next, Dox (1 μM) was mixed with affi-F/AuNPs (3 nM) and incubated at room temperature for 24 h at 37 °C. The mixture was then filtered with 10 kDa MWCO centrifuge filters (Amicon, Millipore, USA) to remove redundant drugs, and the concentrated solution was stored at 4 °C.

### Drug loading study of Dox@affi-F/AuNPs

As mentioned before^36^, the number of FUdR-DNA strands loaded on each F/DNA1-AuNPs can be examined by measuring the concentration of gold nanoparticles and the concentration of FAM-labeled F/DNA1 in each sample. The concentration of gold nanoparticles was determined by UV–Vis spectroscopy, and according to Beer’s law (A = εbc), their absorbance values were related to the nanoparticle concentration. The wavelength of the absorbance maxima (λ) and extinction coefficients (ε) used for 16 nm gold nanoparticles are as follows: λ = 520 nm, ε = 2.67 × 10^8^ M^−1^ cm^−1^^37^. To determine the concentration of fluorescent DNA, F/DNA1-AuNPs were treated with DTT (1.0 M) in PBS, and F/DNA1 were cleaved from the gold nanoparticle surface into solution during an overnight incubation. After removing the gold precipitate by centrifugation, the concentration of F/DNA1 in the supernatant was calculated according to the corresponding standard curve. The number of F/DNA1 attached to each gold nanoparticle was calculated by dividing the concentration of fluorescent DNA by the concentration of gold nanoparticles. Therefore, according to the principle of base complementary pairing, each affi-F/AuNPs contained the same mole amount of affi-F/DNA hybrid strand as F/DNA1, and 20 FUdR molecules were integrated in each affi-F/DNA hybrid strand by covalent bonds.

To test the Dox loading on the affi-F/DNA hybrid strand, 6 µM Dox was mixed with various concentration of DNA duplexes (0, 0.1, 0.2, 0.3, 0.5, 1.0 µM) in PBS, and the fluorescence of DOX (excitation 495 nm, emission 555 nm) was measured. To determine the encapsulation efficiency (EE) of Dox in Dox@affi-F/AuNPs, Dox (1 µM) was mixed with affi-F/AuNPs (3 nM) in PBS, and incubated for 0, 6, 12, 24 and 48 h. The mixture was then centrifuged at 12,000 rpm for 15 min to remove unloaded Dox. From the fluorescence of the removed Dox, the EE of Dox at different incubation times was calculated by back titration.

### In vitro drug release study of Dox@affi-F/AuNPs

To study the Dox release rate, 10 nM Dox@affi-F/AuNPs solution was transferred into a dialysis bag (MWCO 10 kDa) and immersed into PBS (pH 7.4) or acetate buffer (pH 4.5) at 37 °C. The tests were carried out in an incubator shaker (ZWYR-200D, LABWIT Scientific, Shanghai, China) with gentle shaking at a speed of 100 rpm. The fluorescence of Dox in PBS was measured, and the released amount of Dox was calculated at different time intervals. The release rate (R%) of Dox was calculated using the following equation:

$$ {\text{R}}\% \, = {\text{ C}}_{{1}} {\text{/C}}_{0} \times { 1}00 $$

where C_1_ is the concentration of released Dox and C_0_ is the concentration of Dox loaded in Dox@affi-F/AuNPs.

### Nuclease digestion assay

Nucleases are responsible for the degradation of exogenous DNA in vivo, mainly including DNase I and DNase II, which catalyze the hydrolytic cleavage of phosphodiester linkages in DNA backbone. DNase I provides most of the deoxyribonuclease activity in plasma at a level of 0.36 U/mL. While DNase II is a kind of acid endonuclease, and widely exists in animal cells rather than plasma. The stability of affi-F/AuNPs in the plasma was characterized with 3.6 U/mL DNase I solution for 8 h at 37 °C according to the manufacturer's instruction. Subsequently, in order to investigate whether affi-F/AuNPs can be digested after being taken up by cancer cells and release FUdR to inhibit cell proliferation, they were incubated with 20 U/mL DNase II at 37 °C for 8 h. Next, to understand the effect of nucleases on Dox release, Dox@affi-F/AuNPs were treated with DNase I or DNase II for 0, 2, 4, 8, 12 and 24 h. Then, the fluorescence of Dox in the supernatant after centrifugation was measured, and the amount of released Dox was calculated according to the standard curve. The release rate was calculated according to the above formula.

### Transmission electron microscopy (TEM) characterization

The morphology of AuNPs, affi-F/AuNPs and Dox@affi-F/AuNPs were examined by a transmission electron microscope (TEM, H-600, Hitachi, Ltd., Japan). They were diluted with distilled water and placed on a copper electron microscopy grids and negatively stained with a 2% (w/v) phosphotungstic acid solution. The excess fluid was removed with a piece of filter paper, and then dried in air at room temperature. TEM analysis was done for the dried samples^30^.

### Dynamic light scattering (DLS)

The size distributions of AuNPs, affi-F/AuNPs and Dox@affi-F/AuNPs were measured with Nanobrook Omni (Brookhaven Instruments Corporation, USA). The concentration of samples used for DLS analysis was 2 nM^28^.

### Cell cultures

Human breast cancer cell line BT474 and MCF-7 were obtained from cell resource center of Shanghai Biological Sciences Institute (Chinese Academy of Sciences, Shanghai, China). BT474 cells were cultured in Roswell Park Memorial Institute 1640 medium (RPMI-1640, Wisent, China) with FBS and penicillin–streptomycin solution at the concentration of 10% (v/v) and 1% (v/v), respectively. MCF-7 cells were cultured in high-glucose Dulbecco’s modified Eagle’s medium (DMEM, Wisent, China) containing 10% (v/v) FBS and 1% (v/v) penicillin–streptomycin solution. The cells were placed at 37 °C in a humidified atmosphere containing 5% CO_2_^28^.

### Cellular uptake evaluation by confocal laser scanning microscopy

BT474 and MCF-7 cells (1 × 10^5^ cells/well) were separately seeded into a laser confocal dish (NEST, Wuxi, China) overnight at 37 °C. FAM-labeled Dox@affi-F/AuNPs (with equivalent amount of 5 µM FAM) were added to the dishes for 2 h. The culture solution was carefully removed and the cells were washed 3 times with ice-cold PBS, and then fixed with 4% formaldehyde for 20 min at room temperature. The fluorescent images were obtained using a Zeiss laser scanning confocal microscope (Zeiss LSM 880, Germany).

### In vitro cytotoxicity

In vitro cytotoxicity assays of breast cancer cell were performed by MTT method. Briefly, exponentially growing BT474 and MCF-7 cells were harvested and plated in 96-well plates at a concentration of 5 × 10^3^ cells/well, respectively. After the cells were incubated at 37 °C for 24 h, the culture medium was replaced with 100 µL of fresh medium containing different concentrations of FUdR, affi-F/AuNPs, Dox, the physical mixture of FUdR and Dox (named Dox/FUdR (1:4)) and Dox@affi-F/AuNPs, and the cells were incubated for an additional 48 h. Afterward, 10 µL of MTT (5 mg/mL) was added to each well and the plates were incubated at 37 °C for 4 h. The supernatant was discarded, and 100 µL of DMSO was added to each well. The absorbance was determined at 570 nm. Data was reported as the mean of three independent experiments, each run in quintuplicate. The dose response graph was plotted by calculating the percent cell viability using the formula below:

$$ {\text{Cell}}\;{\text{viability}}\;(\% ) = \frac{{{\text{OD}}_{{570({\text{Sample}})}} - {\text{OD}}_{{570({\text{Blank}})}} }}{{{\text{OD}}_{{570({\text{Control}})}} - {\text{OD}}_{{570({\text{Blank}})}} }} \times 100\% $$

In addition, the inhibitory concentration causing 50% growth inhibition (IC50 value) of FUdR and Dox alone and in combination was also determined using an online calculator (https://www.aatbio.com/tools/ic50-calculator). The CI value was calculated by Compusyn software (http://www.combosyn.com) provided by professor Chou TC. The molar ratio of Dox and FUdR in Dox/FUdR (1:4) and Dox@affi-F/AuNPs was fixed at 1:4. The CI values < 1, = 1 and > 1 represent synergism, additive and antagonism, respectively.

### Apoptosis analysis by flow cytometry

The cell apoptosis was evaluated by flow cytometry analysis using Annexin V-FITC/PI apoptosis analysis kit (Absin, Shanghai, China) according to the manufacturer’s instruction. Briefly, BT474 and MCF-7cells were seed in 6-well plates and allowed to grow for 24 h, then exposed to a medium containing FUdR, affi-F/AuNPs, Dox, Dox/FUdR (1:4) and Dox@affi-F/AuNPs (a dose equal to 6 μM FUdR or 2 μM Dox) with a subsequent 12 h incubation. The cells were subjected to apoptosis analysis using flow cytometry. Both early apoptotic (Annexin V-FITC^+^/PI^−^) and late apoptotic (Annexin V-FITC^+^/PI^+^) cells were included in cell apoptosis determinations^28^.

### Statistical analysis

All samples were prepared and tested in triplicates or more. Data were presented as mean ± standard deviation (SD). The statistical significance of differences between groups was determined by the Newman-Keuls analysis. The differences were considered significant for *P < 0.05, and highly significant for **P < 0.01 and extremely significant for ***P < 0.001^28^.

## Results and discussion

### Synthesis and characterization of F/DNA1-SH and F/DNA2-affibody

**Figure 1:**
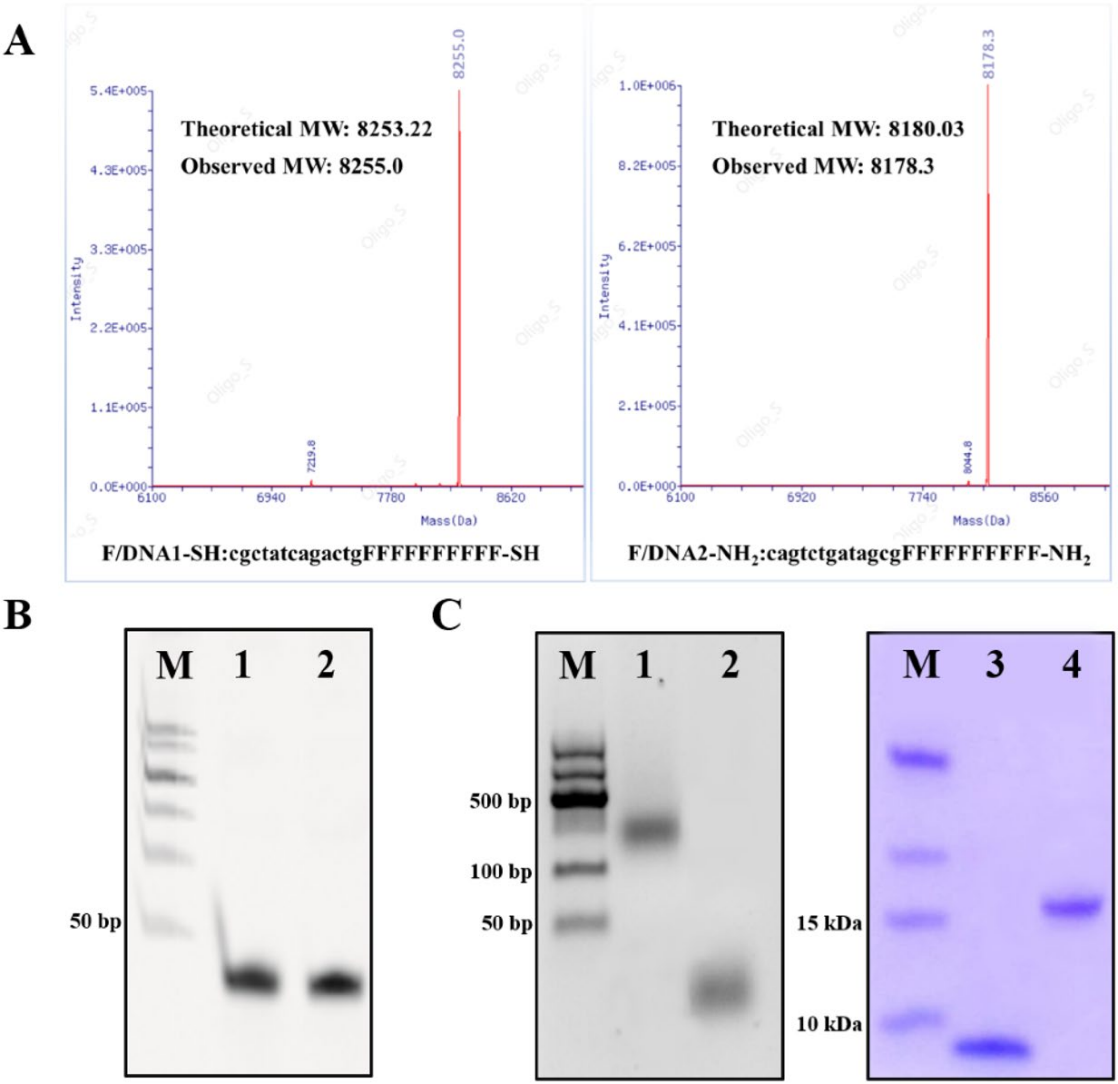
Characterization of F/DNA1-SH, F/DNA2-NH_2_ and F/DNA2-affibody. (**A**) Mass spectrometry of F/DNA1-SH, F/DNA2-NH_2_. (**B**) Denaturing PAGE analysis of F/DNA1-SH and F/DNA2-NH_2_. Lane 1, F/DNA1-SH; Lane 2, F/DNA2-NH_2_. (**C**) Agarose gel and SDS-PAGE analysis of F/DNA2-affibody, F/DNA2-NH_2_ and affibody. Lane 1, F/DNA2-affibody; Lane 2, F/DNA-NH2; Lane 3, affibody; Lane 4, F/DNA2-affibody.

According to our design, FUdR need to be integrated into the two DNA strands. Due to the structural similarity with normal thymidine (T), FUdR were derivatized into its phosphoramidite monomer, and then covalently linked to DNA single strand in the form of phospholipid bonds via DNA solid-phase synthesis technology. Subsequently, a thiol group was modified at the 3′-end of one of the two FUdR-DNA strands (F/DNA1-SH) for self-assembly on the surface of the solid gold sphere through an ‘Au–S’ bonding. An amino group was modified at the 3′-end of the other FUdR-DNA strand to form F/DNA2-NH_2_ for attachment to the targeting ligand affibody molecule. After solid-phase synthesis and purification, the two modified FUdR-DNA strands were identified by mass spectrometry, and the results showed no difference between the measured and theoretical values of their molecular weight (Fig. 1A). In addition, denatured polyacrylamide gel electrophoresis was also used to analyze their properties, and it can be seen from Fig. 1B that the mobility of the two bands was consistent with the designed DNA sequence (Listed in Table S1).

Next, the targeted F/DNA2-affibody was prepared by bioconjugation technology as previously described^28,29^. The affibody molecule Z_hcHER2:342_ was constructed on the basis of Z_HER2:342_, and its C-terminus and N-terminus have been modified with cysteine residue and His-Tag, respectively. Using their respective amino and sulfhydryl group of cysteine, F/DNA2-NH_2_ was bioconjugated with affibody molecule Z_hcHER2:342_ via Sulfo-EMCS-linker to generate F/DNA2-affibody according to Scheme S1. The obtained reaction mixture was subjected to His-tag-mediated affinity chromatography to remove unreacted DNA strands, and the excessive affibody molecules were cleared by anion exchange chromatography (Capto DEAE column)^34^. The pure F/DNA2-affibody conjugate was characterized by agarose gel electrophoresis and SDS-PAGE, respectively. As shown in Fig. 1C, the F/DNA2-affibody (Lane 1) had a slower migration than that of F/DNA2-NH_2_ (Lane 2) because of the attachment of Z_hcHER2:342_. Moreover, it was inferred from the Coomassie blue-stained SDS-PAGE that the molecular weight of F/DNA2-affibody was about 16 kDa, which was much larger than the affibody molecule Z_hcHER2:342_ (~ 8.3 kDa). The above results demonstrated that F/DNA-affibody was successful formed by the combination of F/DNA2-NH_2_ and Z_hcHER2:342._

### Preparation and characterization of affi-F/AuNPs and Dox@affi-F/AuNPs

**Figure 2:**
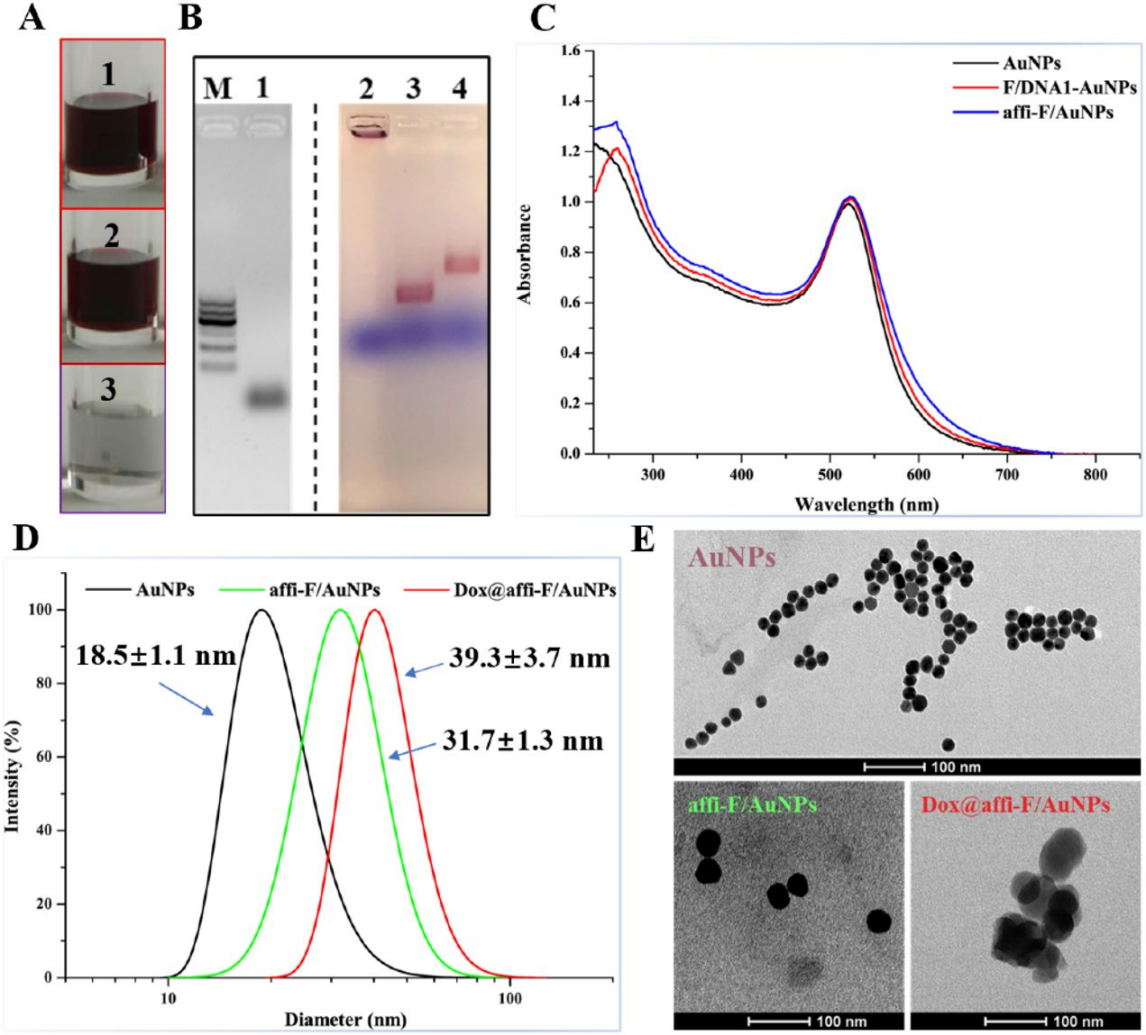
Characterization of affi-F/AuNPs and Dox@affi-F/AuNPs. (**A**) Digital pictures of F/DNA1-AuNPs (1), affi-F/AuNPs (2) and AuNPs (3) after suspension in PBS buffer and overnight storage. (**B**) Agarose gel analysis of affi-F/AuNPs (UV-imaging of gel is on the left of the dividing line, and brightfield imaging of gel is on the right). Lane 1, F/DNA1-SH; Lane 2, AuNPs; Lane 3, F/DNA1-AuNPs; Lane 4, affi-F/AuNPs. (**C**) UV–Vis analysis of AuNPs, F/DNA1-AuNPs and affi-F/AuNPs. (**D**) Particle size distributions of AuNPs, affi-F/AuNPs and Dox@affi-F/AuNPs. (**E**) TEM images of AuNPs, affi-F/AuNPs and Dox@affi-F/AuNPs.

As a nanomaterial hybridized with DNA strands containing drugs, AuNPs were synthesized by the citrate reduction method^35^ with average size of 18.5 nm (Fig. 2D,E). Then, the thiolated F/DNA1-SH strands were conjugated to the citrate-coated AuNPs to form F/DNA1-AuNPs, followed by hybridization with F/DNA2-affibody through the complementary DNA (cDNA) region to construct affi-F/AuNPs. It can be seen from the visual inspection that the successfully synthesized F/DNA1-AuNPs and affi-F/AuNPs maintained good dispersion and the color in solution stayed deep red (Fig. 2A). On the contrary, the non-DNA-functionalized AuNPs aggregated in the buffer and their color changed from light red to light purple. The agarose gel electrophoresis was also used to detect the formation of F/DNA1-AuNPs and affi-F/AuNPs. Due to fluorescence quenching effect of AuNPs^30^, the gel was photographed in bright field after UV-imaging. As shown in Figs. 2B and S4, the mobility of F/DNA1-AuNPs (Lane 3) and affi-F/AuNPs (Lane 4) was much slower than that of F/DNA1-SH (Lane 1), especially affi-F/AuNPs. This was attributed to the enlargement of nanoparticles caused by the coupling of F/DNA1-SH strands with AuNPs and hybridization with F/DNA2-affibody. AuNPs (Lane 2) did not migrate on the gel due to the aggregation of nanoparticle in electrophoresis buffer. Moreover, the results of UV/vis spectra also confirmed the successful synthesis of F/DNA1-AuNPs and affi-F/AuNPs (Fig. 2C).

To load Dox, affi-F/AuNPs solution was mixed with doxorubicin hydrochloride aqueous solution, and the Dox@affi-F/AuNPs mixture was treated by multiple centrifugation-washing procedures to remove free Dox. The obtained products were characterized by dynamic light scattering (DLS) and transmission electron microscopy (TEM), respectively. The particle sizes of affi-F/AuNPs and Dox@affi-F/AuNPs determined by DLS were 31.7 ± 1.3 nm and 39.3 ± 3.7 nm, respectively (Fig. 2D). As shown in the TEM images in Fig. 2E, affi-F/AuNPs and Dox@affi-F/AuNPs were larger and denser spheres than AuNPs. Obviously, there was an adhesive layer on the periphery of Dox@affi-F/AuNPs, which certificated that the affibody-F/DNA hybrid strands (affi-F/DNA) incorporating Dox successfully covered the surface of AuNPs.

### Drug loading and in vitro release analysis of Dox@affi-F/AuNPs

**Figure 3:**
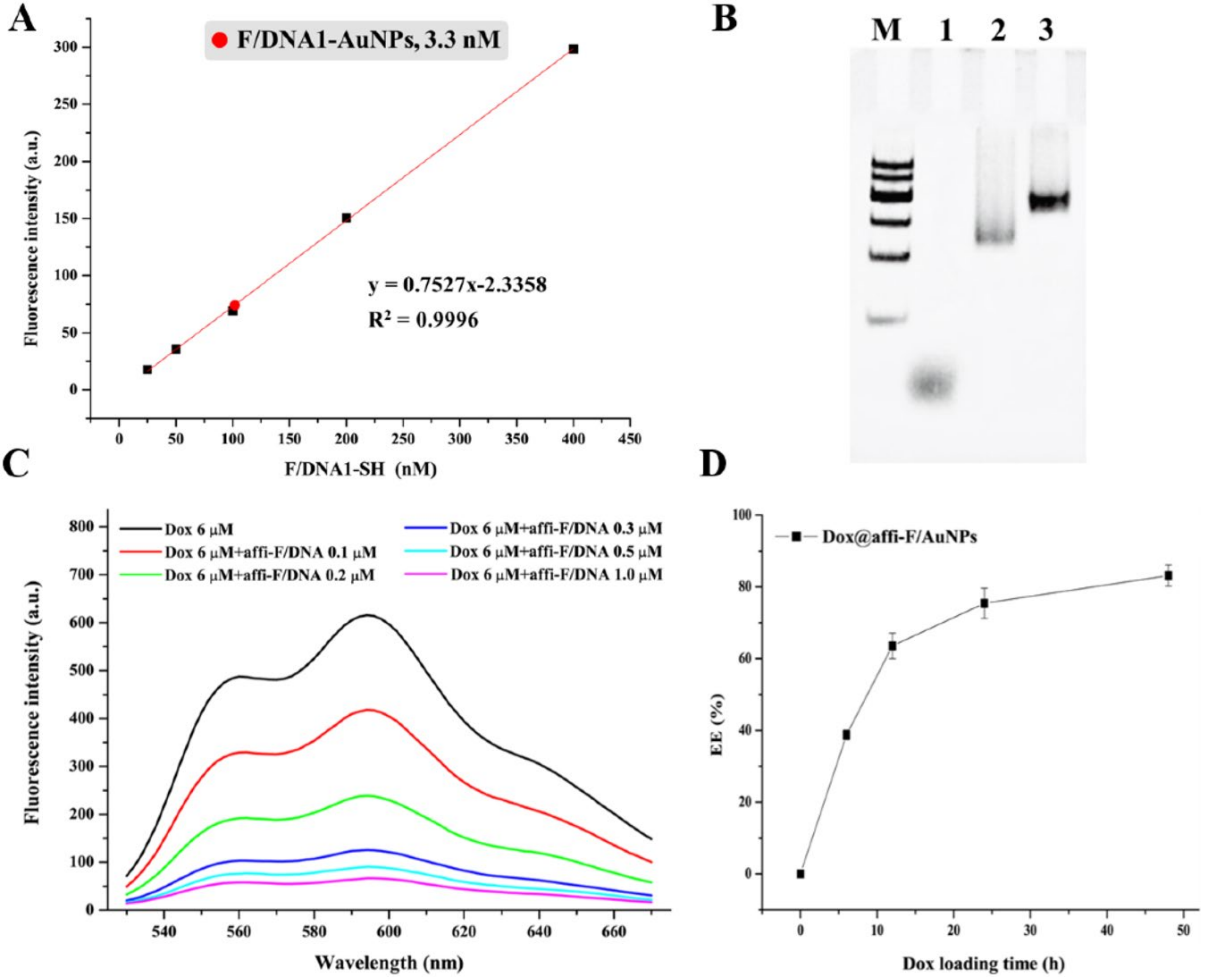
Drug loading analysis of Dox@affi-F/AuNPs. (**A**) DNA quantification of F/DNA1-AuNPs. (**B**) PAGE gel image which prove the duplex formation from affi-F/DNA. Lane 1, F/DNA1-SH; Lane 2, F/DNA2-affibody; Lane 3, affi-F/DNA hybrid strand. (**C**) The fluorescence spectra of Dox with various concentrations of affi-F/DNA (0, 0.1, 0.2, 0.3, 0.5 and 1 μM). (**D**) The encapsulation efficiency (EE) of affi-F/AuNPs to Dox with different incubation time (0, 6, 12, 24 and 48 h).

In order to determine the drug loading of FUdR in Dox@affi-F/AuNPs, the amount of affi-F/DNA was detected by measuring the fluorescence of FAM labeled on F/DNA1-SH. To eliminate fluorescence quenching effect of AuNPs, the F/DNA1-SH fixed on the surface of gold nanoparticle was chemically released from F/DNA1-AuNPs by an exchange reaction with dithiothreitol (DTT), and the concentration of fluorescent F/DNA1-SH was measured as described previously for DNA-AuNPs^36^. According to the standard curve, 31 F/DNA1-SH strands were modified on a single F/DNA1-AuNPs (Fig. 3A). Based on the principle of DNA hybridization, each affi-F/AuNPs contained 31 affi-F/DNA, and loaded 806 FUdR molecules (Scheme 1A). Then, the loading amount of anti-cancer drug Dox was investigated by monitoring the fluorescence intensity. As Dox was loaded into the DNA duplexes of affi-F/AuNPs by intercalating, the formation of duplexes was first confirmed by polyacrylamide gel electrophoresis (Fig. 3B). The fluorescence spectrum of Dox (Fig. 3C) showed that its fluorescence was quenched gradually with the increase of DNA duplexes concentration, which indicated that Dox could be loaded into affi-F/DNA effectively. Afterward, the assessment of encapsulation efficiency (EE) of affi-F/AuNPs to Dox revealed that 63.5% of Dox (1 µM) was loaded into affi-F/AuNPs (3 nM) after 12 h of incubation, and each Dox@affi-F/AuNPs contained 211 Dox molecules (Fig. 3D).

**Figure 4:**
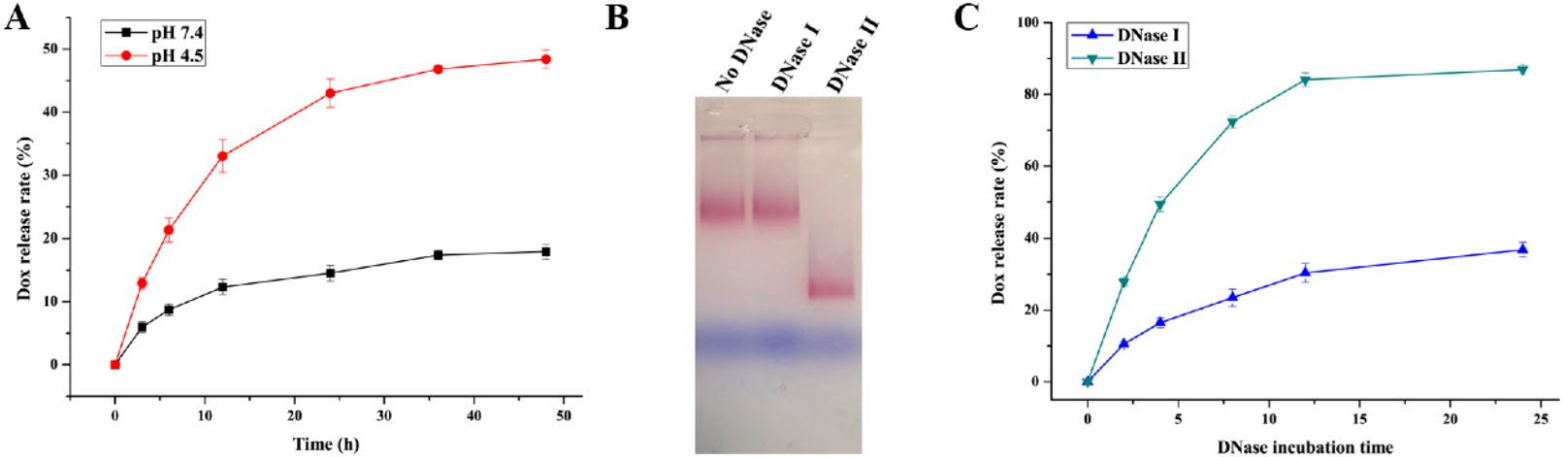
In vitro drug release behavior and nuclease digestion analysis of Dox@affi-F/AuNPs. (**A**) Dox release profile of Dox@affi-F/AuNPs in PBS (pH 7.4) and acetate buffer (pH 4.5). (**B**) Digestion analysis of affi-F/AuNPs by DNase I and DNase II. (**C**) Dox release from Dox@affi-F/AuNPs in DNase I and DNase II solution.

Dox and FUdR release behaviors were evaluated in the buffers of pH 7.4 (approximate pH of blood) and pH 4.5 (approximate pH of acidic endosomes), as well as DNase solutions. As shown in Fig. 4A, Dox@affi-F/AuNPs did not show significant Dox release (17.9%) in PBS at pH 7.4, while a time-dependent release behavior was observed in the acetate buffer at pH 4.5, and the drug release rate reached 48.4% after 48 h. These results suggested that Dox@affi-F/AuNPs could help reduce the toxicity of Dox to normal tissues at the physiological pH value of the human body (pH 7.4)^38^. Moreover, the enhanced release efficiency under acidic conditions might initiate rapid release of Dox from Dox@affi-F/AuNPs after HER2-mediated internalization^39^. Since FUdRs were covalently integrated into the DNA strands via phospholipid bonds, no free FUdR molecules were detected in either of the above buffers. Subsequently, the in vitro drug release behavior of nuclease-treated affi-F/AuNPs and Dox@affi-F/AuNPs was analyzed. After treated with different DNase for 8 h, it was found that affi-F/AuNPs had different migration positions (Fig. 4B). The mobility of DNase I-treated affi-F/AuNPs was roughly equivalent to that of gold particles which were not treated with DNase, indicating that they could be stable during in vivo transportation process without being degraded by DNase I in the blood. Compared with control, affi-F/AuNPs treated with DNase II migrated much faster, which might be due to the significant degradation of DNA strands containing FUdRs that made gold nanoparticles smaller. The result also suggested that after affi-F/AuNPs entered into cancer cells, FUdR molecules could be released through the DNA degradation caused by DNase II, which was widely present in mammalian cells. Figure 4C displayed the Dox release behavior in Dox@affi-F/AuNPs after destruction by DNase I and DNase II. When incubated with 3.6 U/mL DNase I (10 times plasma level)^40^ at 37 °C for 24 h, Dox@affi-F/AuNPs only released 36.8% of Dox, supporting that the dense spherical DNA-AuNPs had excellent resistance to enzymatic nucleic acid degradation in plasma^41^. In contrast, after incubated with 20 U/mL DNase II at 37 °C for 24 h, the DNA duplexes of affi-F/AuNPs were destroyed and resulted in 86.9% Dox release (Fig. 4C). The dual drugs release based on DNase II degradation could allow Dox@affi-F/AuNPs to fully exert their synergistic effect after being internalized into cancer cells, thereby inhibiting tumor growth.

### Targeted cellular uptake of Dox@affi-F/AuNPs

**Figure 5:**
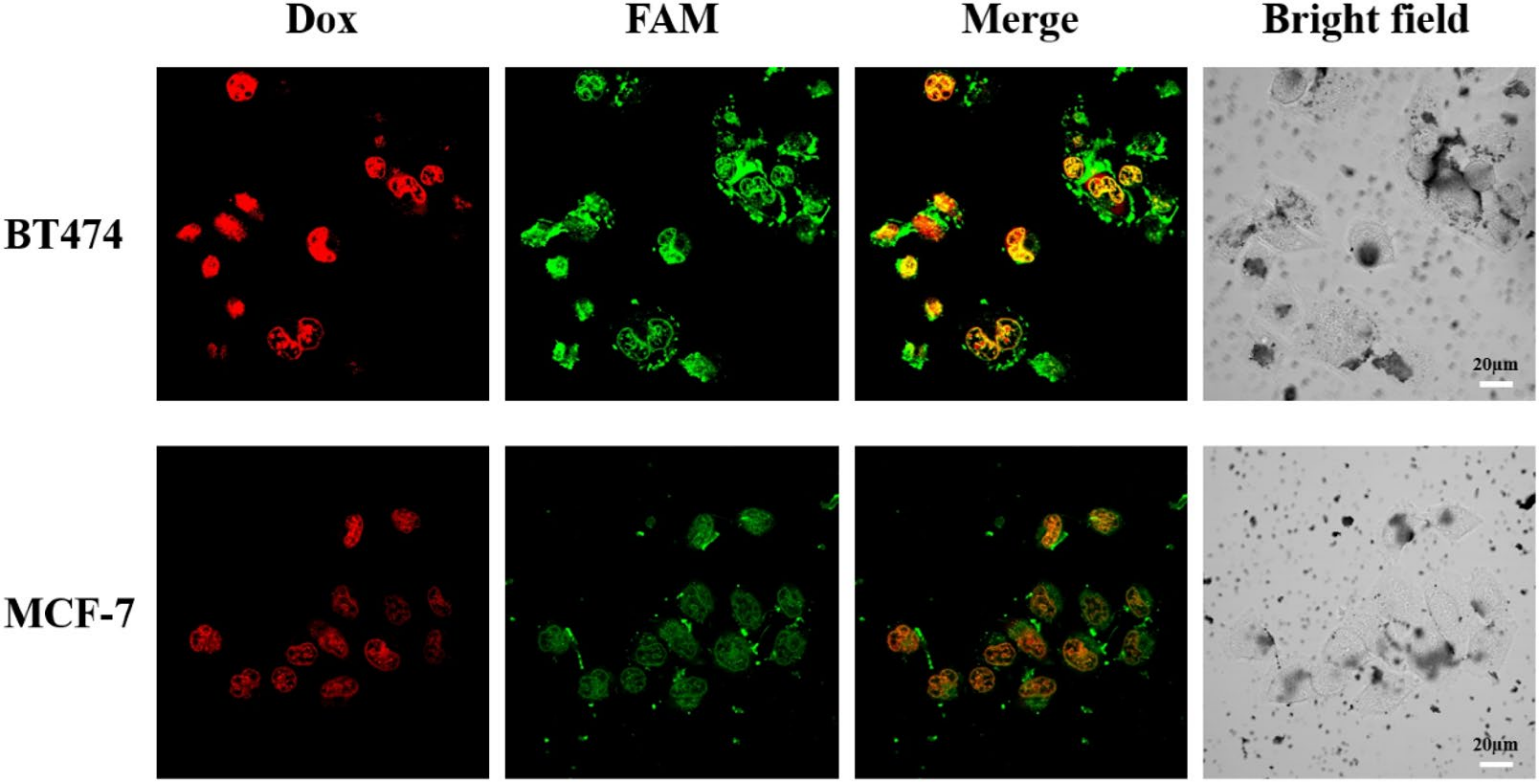
LSCM images of BT474 and MCF-7 cells incubated with Dox@affi-F/AuNPs. BT474 and MCF-7 cells were treated with FAM-labelled Dox@affi-F/AuNPs for 2 h (relative FAM = 5 µM). The red fluorescence was arisen from Dox (ex/em, 488/575 nm), and the green fluorescence was arisen from FAM (ex/em, 494/522 nm). Scale bar: 20 µm.

Affibody molecule Z_hcHER2:342_, as a substitute of antibodies, can specifically bind HER2 and exhibits higher affinity than antibodies^42^. Therefore, the targeted cell uptake of Dox@affi-F/AuNPs was evaluated using BT474 and MCF-7 cells, which were identified as breast cancer cells with overexpressed HER2 and low HER2 expression, respectively, by western blot (Fig. S1). To investigate the cellular uptake efficiency of Dox and FUdR, Dox@affi-F/AuNPs were incubated with BT474 and MCF-7 cells for 2 h at 37 °C and analyzed by laser scanning confocal microscopy (LSCM). Dox can be visualized with its own red fluorescence. Once it was released from Dox@affi-F/AuNPs, the intracellular uptake of Dox can be directly detected by LSCM. Since FUdR itself did not produce fluorescence, FAM was labeled on F/DNA1-SH that was coupled to the gold nanoparticles via Au–S coordination bonds. As shown in Fig. 5, there was a stronger green fluorescence within the BT474 cells than that within the MCF-7 cells. It was inferred that the overexpressed HER2 resulted in more Dox@affi-F/AuNPs entering BT474 cells through receptor-mediated cell internalization, while the HER2 low-expressing cancer cells could not perform this function. Importantly, the accumulation of Dox in BT474 cells was much higher than that in MCF-7 cells, which further confirmed that Dox@affi-F/AuNPs could be an effective targeting nanocarrier for selective delivery of Dox to HER2 overexpressed cancer cells, thus reducing the toxic and side effects of drugs on normal tissues. Specifically, the targeted intake capacity of Dox@affi-F/AuNPs for the HER2 overexpressing BT474 cells was about two-fold higher than that for the HER2 low-expressing MCF-7 cells (Fig. S2). Together, these results manifested that the newly established DNA-gold nanoparticles had excellent targeting property and could simultaneously and efficiently deliver dual drugs (Dox and FUdR) into HER2 overexpressing cancer cells.

### In vitro cytotoxicity and synergistic effect

**Figure 6:**
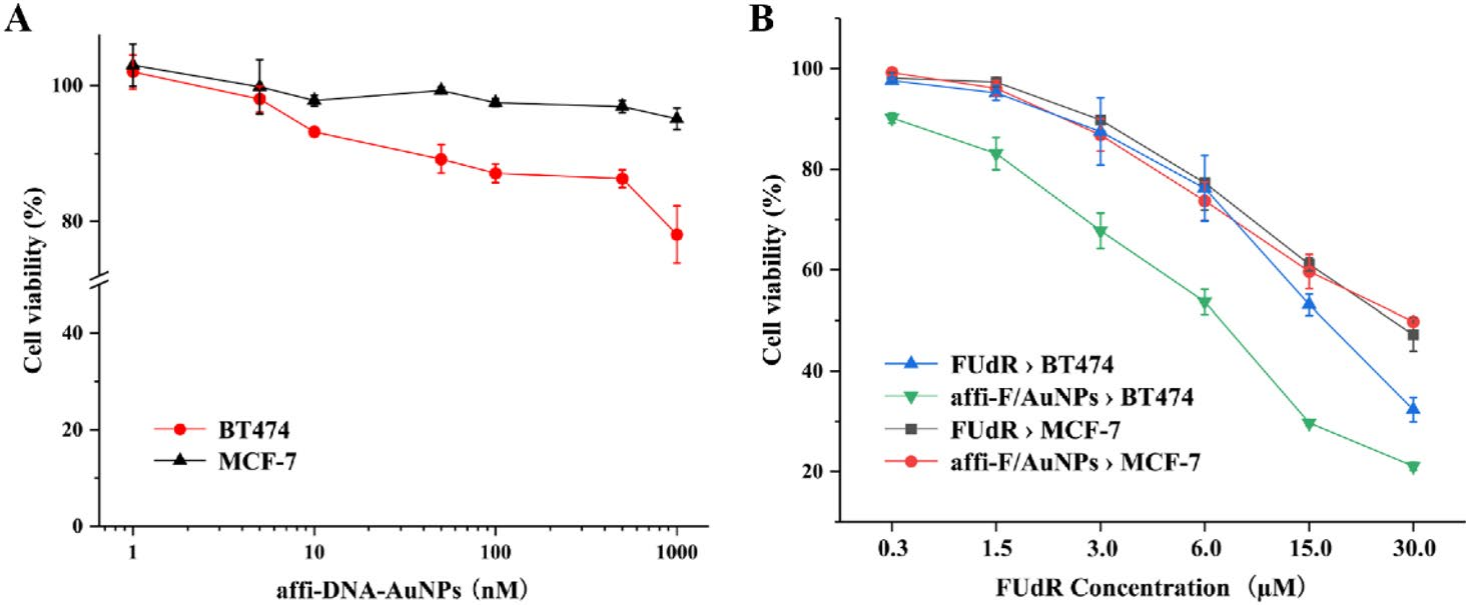
Cytotoxicity assay of affi-DNA-AuNPs and affi-F/AuNPs. (**A**) Cell viability of BT474 and MCF-7 cells incubated with different concentrations of affi-DNA-AuNPs. (**B**) Cell viability of BT474 and MCF-7 cells treated with different concentrations of affi-F/AuNPs.

**Figure 7:**
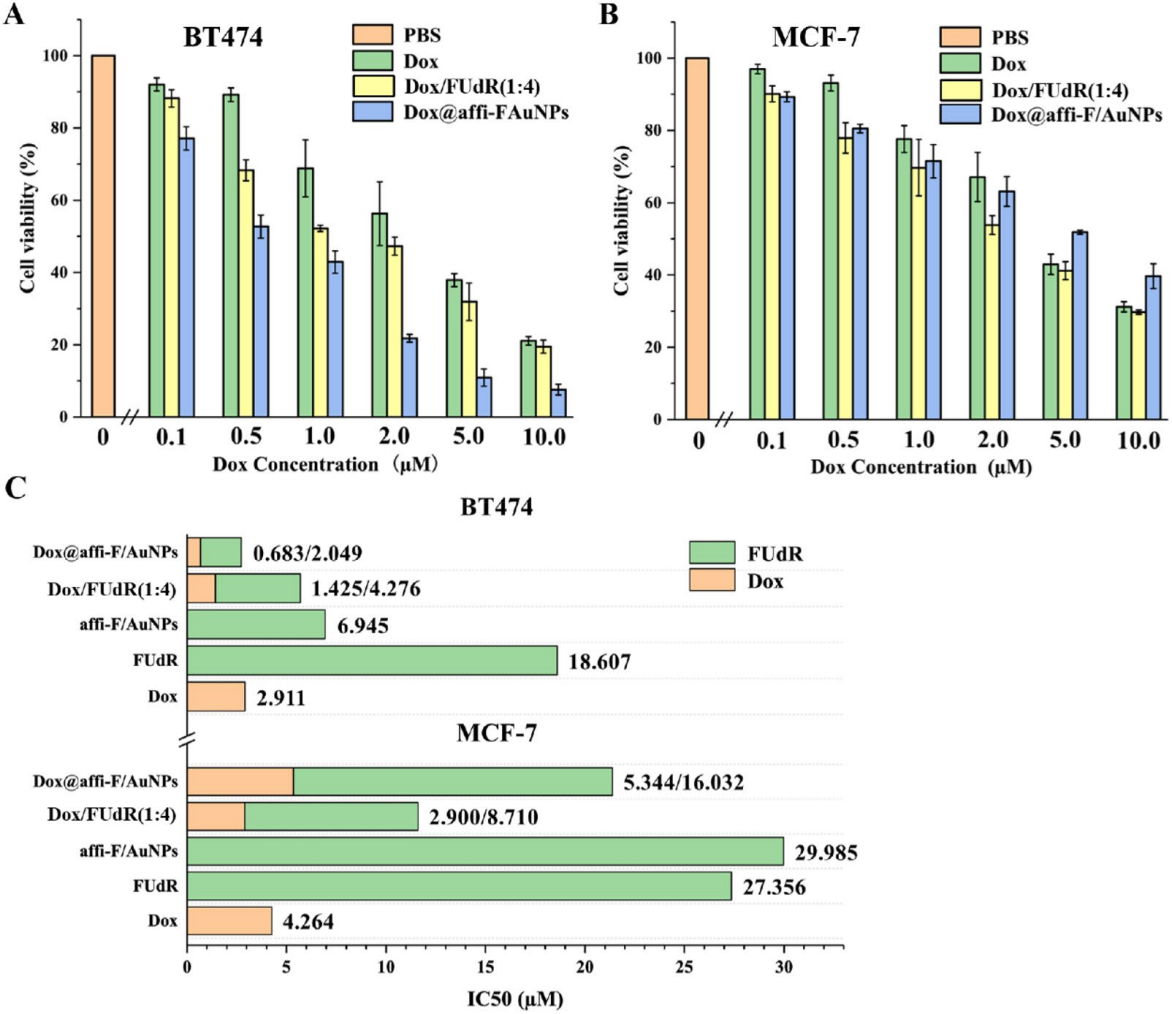
Cytotoxicity assay. Cell viability of BT474 (**A**) and MCF-7 (**B**) incubated with different concentrations of Dox, Dox/FUdR(1:4) and Dox@affi-DNA-AuNPs. (**C**) IC50 values of FUdR and Dox in BT474 and MCF-7 cells.

Firstly, the selective cytotoxicity of affi-F/AuNPs for breast cancer cells was investigated by MTT method. The extensive studies of DNA-AuNPs have proven that they have no apparent cytotoxicity and little innate immune response^43,44^, and the assays of affi-DNA-AuNPs without FUdR showed that they had little cytotoxic profile in BT474 and MCF-7 cells even after 48 h of incubation (Fig. 6A). After loaded with FUdR, affi-F/AuNPs displayed obvious cytotoxicity in both breast cancer cells (Fig. 6B). However, compared to MCF-7 cells, affi-F/AuNPs caused higher cytotoxicity in BT474 cells, and its IC50 value was much lower than that of MCF-7 cells (6.95 µM vs 29.98 µM, Fig. 7C). This may be due to the affibody-mediated selective inhibition of HER2 overexpressing cancer cells.

Subsequently, the cytotoxicity of free drug combination of Dox and FUdR (Dox/FUdR (1:4)), and their combination in DNA-gold nanoparticles (Dox@affi-F/AuNPs) was also evaluated using BT474 and MCF-7 cells. As shown in Fig. 7A and B, more cancer cells were killed by drug combination than any drug alone at each concentration, and the inhibition rate was increased with the increase of drug concentration whether it was a single drug or a combination. In particular, Dox@affi-F/AuNPs, as targeted gold nanoparticles co-loaded with Dox and FUdR, exhibited higher inhibitory efficiency on BT474 cells than the simple mixture of the two drugs, which indicated that the targeted nanocarriers greatly increased cellular uptake of both drugs by HER2 overexpressing cancer cells. However, the toxicity of Dox@affi-F/AuNPs to MCF-7 cells was lower than that of free combination, which may be related to the inability of HER2 low-expressing cells to perform affibody-mediated receptor endocytosis^39^. The IC50 values of different drug combinations also confirmed the above results (Fig. 7C).

**Figure 8:**
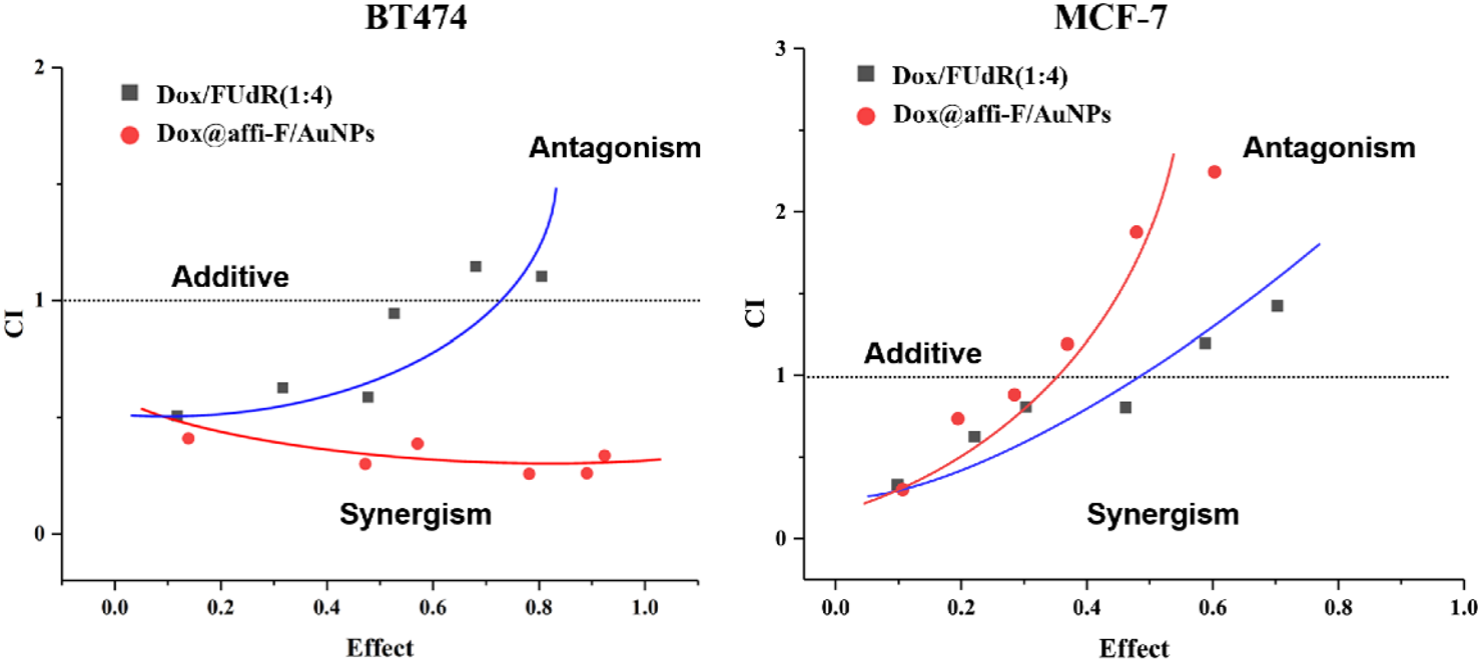
Synergetic effect analysis of Dox/FUdR (1:4) and Dox@affi-F/AuNPs in BT474 and MCF-7 cells. The combination index (CI) value was calculated by Compusyn software (http://www.combosyn.com). The CI values greater than 1, equal to 1 and less than 1 are considered as antagonistic, additive and synergistic effects, respectively.

In order to determine whether the combination of Dox and FUdR interact with each other in antagonistic, additive or synergistic manner, combination index (CI) analysis was conducted based on Chou-Taladay method^45^. The CI value was calculated by Compusyn software (http://www.combosyn.com) provided by professor Chou TC. The CI values greater than 1, equal to 1 and less than 1 are considered as antagonistic, additive and synergistic effects, respectively^46^. Figure 8 showed that free drug combination had a synergistic killing effect on both breast cancer cells in low-inhibition efficacy areas, but had antagonism in high-inhibition efficacy areas, which was not ideal result because high inhibitory efficacy was usually the major consideration in treating cancer^47^. Notably, Dox@affi-F/AuNPs exhibited strong synergism in both low and high-inhibition areas in the treatment of HER2 overexpressing BT474 cells, which suggested that gold nanoparticles can provide a relatively precise drug ratio (1:4 for Dox to FUdR) within cancer cells, and ensure a wider synergistic range.

### Cell apoptosis assays

**Figure 9:**
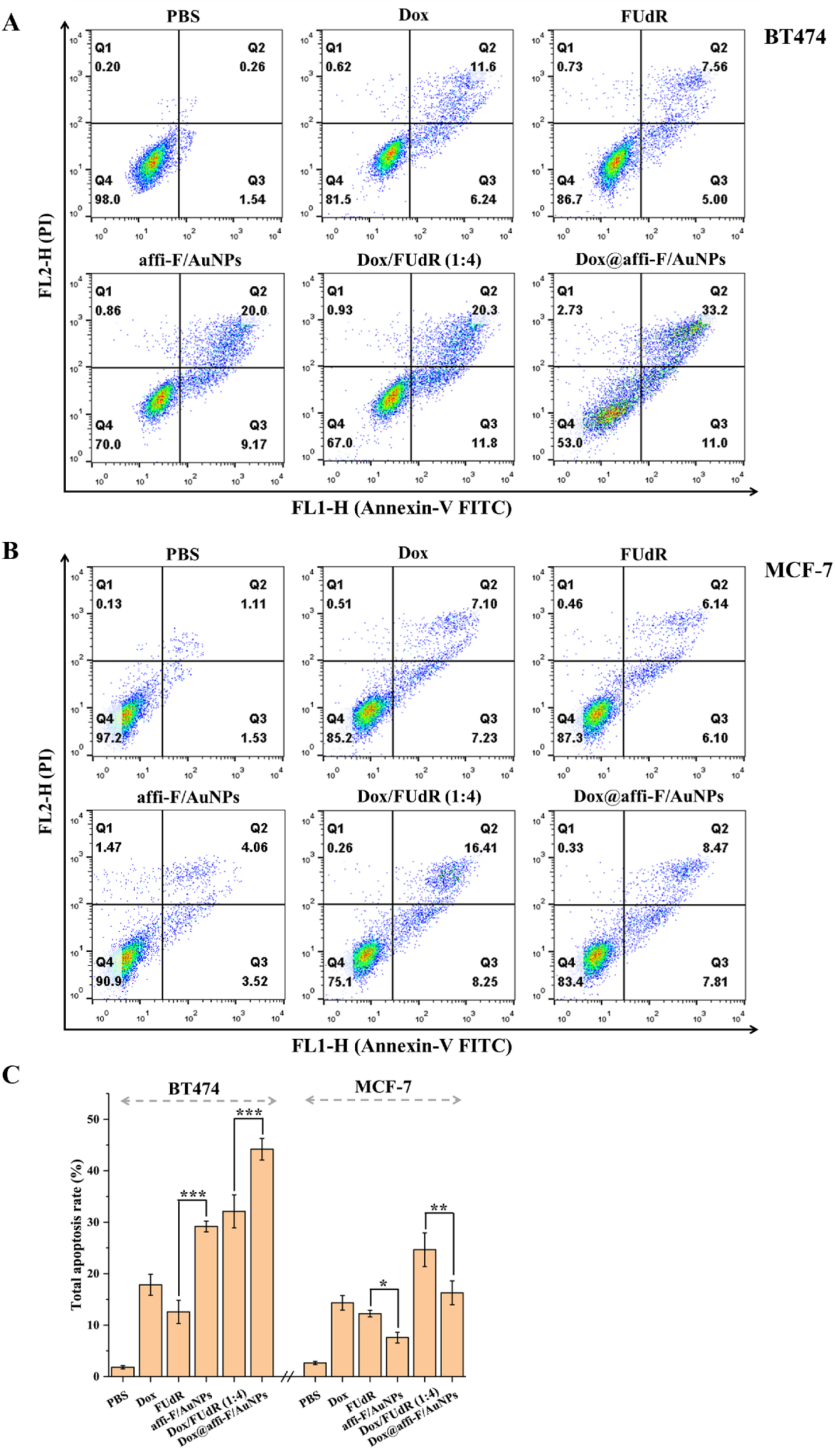
Cell apoptosis assay using flow cytometry. (**A**,**B**) Flow cytometry scatterplots of apoptosis rate in BT474 and MCF-7 cells. (**C**) Quantitative analysis of the total apoptosis rate in above cells.

In order to understand the mechanism of enhanced antitumor activity by Dox@affi- F/AuNPs, the cell apoptosis assays were therefore carried out by Annexin V-FITC/propidium iodide (PI) double staining. BT474 and MCF-7 cells were pretreated with Dox (2 µM), FUdR (6 µM), affi-F/AuNPs, Dox/FUdR(1:4) and Dox@affi-F/AuNPs (a dose equal to 2 μM Dox and 6 μM FUdR) for 12 h, and the apoptosis results were displayed in Fig. 9. The single use of free Dox and FUdR induced a low apoptosis rate in BT474 and MCF-7 cells. However, affi-F/AuNPs treated BT474 cells exhibited significant increased apoptosis rate of 29.2%. By contrast, the apoptosis rate of MCF- 7 cells treated with affi-F/AuNPs was only 7.58%, which was lower than that treated with free FUdR. These results indicated that the targeting ability of affibody can lead to significant differences in the uptake of drug loaded gold nanoparticles by cells with different HER2 expression levels. Based on this, the drug combination in affibody modified gold nanoparticles (Dox@affi-F/AuNPs) also caused an obvious difference in the apoptosis level between BT474 and MCF-7 cells. In BT474 cells, Dox@affi-F/AuNPs resulted in a higher apoptosis rate than the simple mixture of two drugs, and their apoptosis rate were 44.2% and 32.1%, respectively. However, Dox @ affi-F/AuNPs showed opposite results in MCF-7 cells, which may be more beneficial to reduce the toxic side effects of nanodrugs. The results also confirmed that the Dox and FUdR in gold nanoparticles had a remarkable synergistic effect on HER2 overexpressed cancer cells via a mechanism of enhanced apoptosis.

## Conclusions

Based on the structural similarity between nucleoside analog therapeutics and natural nucleobases, an affibody modified DNA-AuNPs (affi-F/AuNPs) nanomedicine platform integrated with FUdRs was successfully constructed for the first time. Using the capability of doxorubicin to intercalate DNA duplexes, the new dual-drug-containing DNA-AuNPs, Dox@affi-F/AuNPs, were prepared, and the co-loading of Dox and FudR was realized. The newly constructed DNA-AuNPs exhibited excellent stability in simulated physiological conditions. Moreover, Dox@affi-F/AuNPs had the ability of targeting due to the presence of affibody, which caused specific uptake of the dual-loaded drug gold nanoparticles by HER2 overexpressing cancer cells. When the nano-drugs entered the cell, DNase II triggered the degradation of exogenous DNA, which promoted the synchronous release of FUdR and Dox from Dox@affi-F/AuNPs. In vitro cytotoxicity test not only confirmed that affi-F/AuNPs had selective inhibition effect on cancer cells with different HER2 expression levels, but also proved that Dox@affi-F/AuNPs produced a higher synergistic killing activity than the simple mixture of Dox and FUdR on HER2 overexpressing breast cancer cells. Furthermore, the related mechanistic studies allowed us to understand that Dox@affi-F/AuNPs achieved a remarkable combined antitumor activity of Dox and FUdR by promoting more cells to enter apoptosis pathway. Therefore, our work provided a new strategy for combined drug loading based on affibody-DNA-AuNPs to achieve targeted and synergistic treatment of two or even multiple chemotherapeutic drugs.

## Supplementary Information

Supplementary information.
