# Supplementary material for: Co-delivery of 5-fluorodeoxyuridine and doxorubicin via gold nanoparticle equipped with affibody-DNA hybrid strands for targeted synergistic chemotherapy of HER2 overexpressing breast cancer
Source: Sci Rep. 2020 Dec 16;10:22015. doi: 10.1038/s41598-020-79125-0 (PMC7745031; doi:10.1038/s41598-020-79125-0)
Supplement: Supplementary file 1 — Supplementary information. [file 41598_2020_79125_MOESM1_ESM.docx]

Supporting Information

**Co-delivery of 5-fluorodeoxyuridine and doxorubicin *via* gold nanoparticle equipped with affibody-DNA hybrid strands for targeted synergistic chemotherapy of HER2 overexpressing breast cancer**

Chao Zhang, ^§^ Fanghua Zhang, ^§^ Mengnan Han,Xuming Wang, Jie Du, Honglei Zhang^*^ and Wei Li^*^

College of Chemistry and Environmental Science, Key Laboratory of Chemical Biology of Hebei Province, Laboratory of Medicinal Chemistry and Molecular Diagnosis of the Ministry of Education, Hebei University, Baoding 071002, China

§These authors contributed equally to this work

Correspondence: Honglei Zhang; Wei Li
Tel +86 312 592 9009

Fax +86 312 592 9009
E-mail: zhanghonglei@hbu.edu.cn; liweihebeilab@163.com

Table S1. Detailed information of the ssDNA sequence

| ssDNA | Sequence |
| --- | --- |
| F/DNA1-SH | 5’-cgctatcagactgFFFFFFFFFF-SH-3’ |
| F/DNA2-NH_2_ | 5’-cagtctgatagcgFFFFFFFFFF-NH_2_-3’ |
| DNA1 | 5’-cgctatcagactgTTTTTTTTTT-SH-3’ |
| DNA2 | 5’-cagtctgatagcgTTTTTTTTTT-NH_2_-3’ |
| FAM-F/DNA1-SH | 5’-FAM/cgctatcagactgFFFFFFFFFF-SH-3’ |

5-Fluorodeoxyuridine is denoted as F.

**
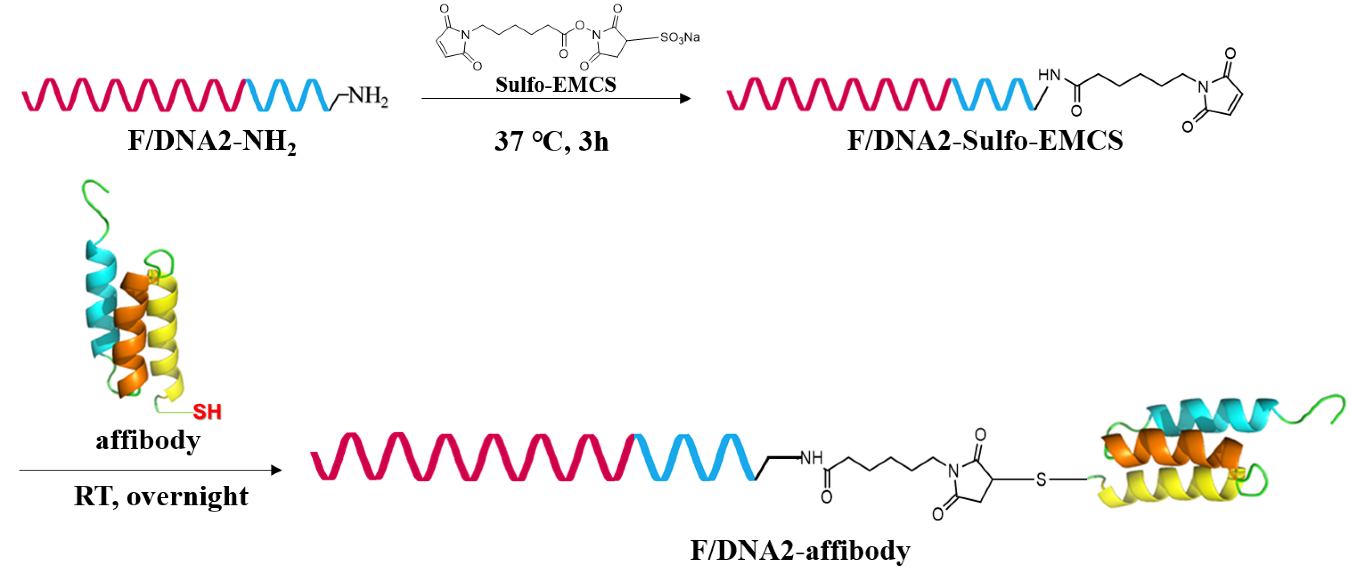
Scheme S1.** Strategy for preparing F/DNA2-affibody


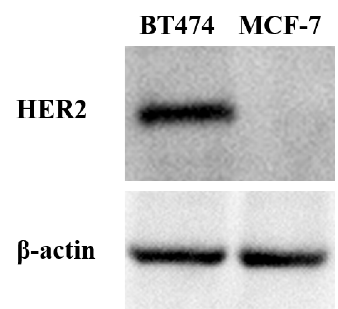


**Figure S1.** Western blot analysis of HER2 expression levels of BT474 and MCF-7 cells


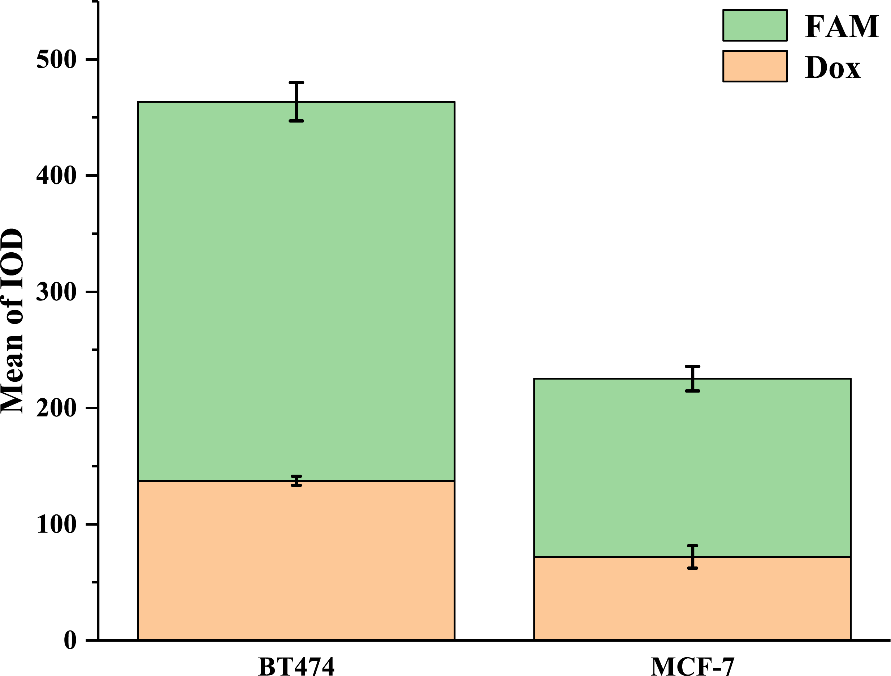


**Figure S2.** Fluorescence intensity analysis in BT474 and MCF-7 cells by Image-Pro Plus software

**
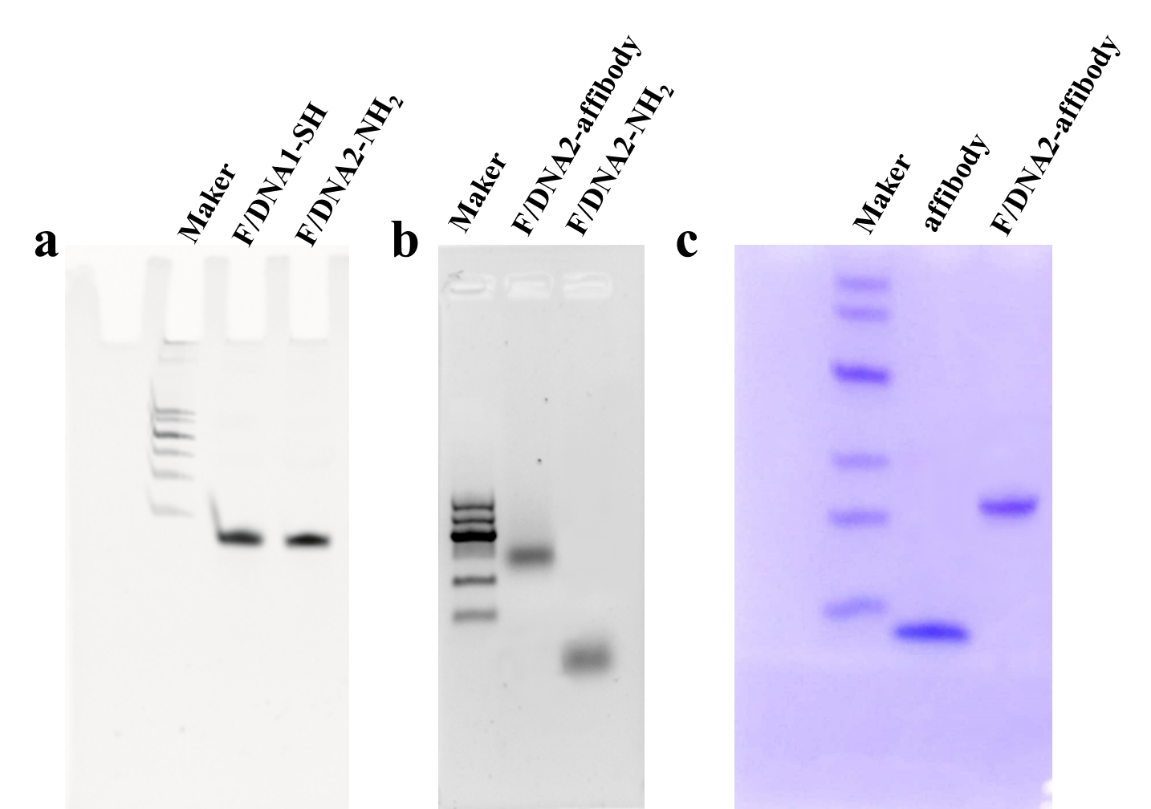
**

**Figure S3.** This figure shows full-length gels in the corresponding panels of Figure 1. (a) Denaturing PAGE analysis of F/DNA1-SH and F/DNA2-NH_2_. (b) Agarose gel analysis of F/DNA2-affibody, F/DNA2-NH_2_ and affibody. (c) SDS-PAGE analysis of affibody and F/DNA2-affibody.


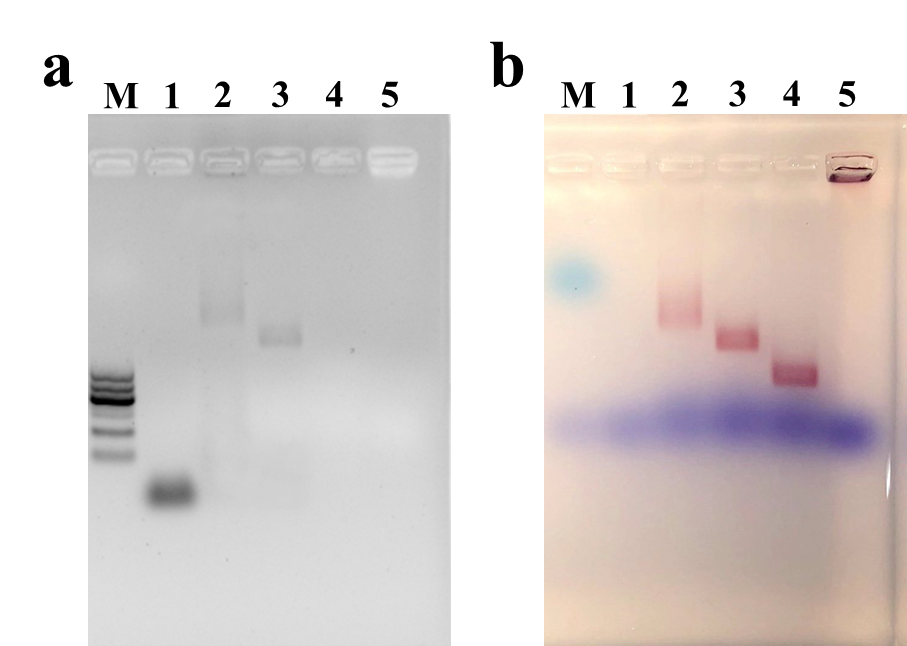


**Figure S4.** This figure shows full-length gels in the corresponding panels of Figure 2. (a) Agarose gel analysis of affi-F/AuNPs and Dox@affi-F/AuNPs (UV-imaging). Lane 1, F/DNA1-SH; Lane 2, Dox@affi-F/AuNPs; Lane 3, affi-F/AuNPs; Lane 4, F/DNA1-AuNPs; Lane 5, AuNPs. (b) Agarose gel analysis of affi-F/AuNPs and Dox@affi-F/AuNPs (Digital imaging). Lane 1, F/DNA1-SH; Lane 2, Dox@affi-F/AuNPs; Lane 3, affi-F/AuNPs; Lane 4, F/DNA1-AuNPs; Lane 5, AuNPs.
